# Liquid–Liquid Phase Separation by Intrinsically Disordered Protein Regions of Viruses: Roles in Viral Life Cycle and Control of Virus–Host Interactions

**DOI:** 10.3390/ijms21239045

**Published:** 2020-11-28

**Authors:** Stefania Brocca, Rita Grandori, Sonia Longhi, Vladimir Uversky

**Affiliations:** 1 Department of Biotechnology and Biosciences, University of Milano-Bicocca, 20126 Milano, Italy; 2 Laboratoire Architecture et Fonction des Macromolécules Biologiques (AFMB), Aix-Marseille University and CNRS, 13288 Marseille, France; 3 Department of Molecular Medicine, Byrd Alzheimer’s Research Institute, Morsani College of Medicine, University of South Florida, Tampa, FL 33601, USA; 4 Laboratory of New Methods in Biology, Institute for Biological Instrumentation of the Russian Academy of Sciences, Federal Research Center “Pushchino Scientific Center for Biological Research of the Russian Academy of Sciences”, 142290 Pushchino, Russia

**Keywords:** intrinsically disordered proteins, viruses, liquid condensates, phase separations and transitions, membrane-less organelles, virus–host cell interactions

## Abstract

Intrinsically disordered proteins (IDPs) are unable to adopt a unique 3D structure under physiological conditions and thus exist as highly dynamic conformational ensembles. IDPs are ubiquitous and widely spread in the protein realm. In the last decade, compelling experimental evidence has been gathered, pointing to the ability of IDPs and intrinsically disordered regions (IDRs) to undergo liquid–liquid phase separation (LLPS), a phenomenon driving the formation of membrane-less organelles (MLOs). These biological condensates play a critical role in the spatio-temporal organization of the cell, where they exert a multitude of key biological functions, ranging from transcriptional regulation and silencing to control of signal transduction networks. After introducing IDPs and LLPS, we herein survey available data on LLPS by IDPs/IDRs of viral origin and discuss their functional implications. We distinguish LLPS associated with viral replication and trafficking of viral components, from the LLPS-mediated interference of viruses with host cell functions. We discuss emerging evidence on the ability of plant virus proteins to interfere with the regulation of MLOs of the host and propose that bacteriophages can interfere with bacterial LLPS, as well. We conclude by discussing how LLPS could be targeted to treat phase separation-associated diseases, including viral infections.

## 1. Introduction to Intrinsically Disordered Proteins (IDPs)

The discovery that intrinsically disordered proteins (IDPs), i.e., functional proteins devoid of stable secondary and/or tertiary structure, are ubiquitous and widespread in the protein realm has triggered a paradigm shift in biochemistry [1,2,3,4,5,6,7,8]. Like Copernican revolution, this call to a dramatic change in the way proteins should be looked at has engendered some resistance in the scientific community. This intellectual reluctance is probably tied to the difficulty of integrating/reconciling the notion of structural disorder with the overwhelming examples of well-structured proteins deposited in the protein data bank (PDB). Paradoxically, however, the development of the field of structural disorder has benefited from the spectacular development of structural biology, especially in the post-genomic era. The continuously growing number of solved structures deposited in the PDB has been instrumental to the development of disorder predictors, most of which have been trained to recognize ordered and disordered regions, typically represented by “regions of missing electron density” in PDB files [9,10]. 

IDPs were, and are still, often considered as proteins defying the so-called “structure-function” paradigm, which posits that the function of proteins is dictated by their structure [11]. However, IDPs only defy the notion that a unique and precise 3D structure is required for function, while they do not challenge the notion that the structure is deeply interconnected with function. IDPs are unable to fold into a precise 3D structure and rather exist as highly dynamic and heterogeneous conformational ensembles. This high conformational heterogeneity is required for their function. Therefore, as for “classical” (i.e., folded) proteins, the function of IDPs stems from their peculiar structure and, obviously, IDPs do not transgress thermodynamic rules. Accordingly, the “structure-function” paradigm should be revised and extended to yield a new paradigm referred to as “ensemble structure-function” or “dynamics-function relation”. Protein dynamics has long been recognized as a major determinant of protein activity (e.g., induced fit in protein–protein interactions, allosteric regulation of protein activity, transport processes, etc.). Increasing efforts are continuously devoted to method development for probing protein dynamics from the experimental and computational perspectives [12]. 

The failure to adopt a unique 3D structure under physiological conditions is encoded in the amino acid sequence, which designs a flat energy landscape, rather than the folding funnel of ordered proteins [13]. IDPs have, in fact, a biased sequence composition, being enriched in polar and charged residues (referred to as “disorder promoting residues”) and depleted in hydrophobic and bulky residues (referred to as “order promoting residues”) [14,15]. 

Because of their enrichment in charged residues, IDPs can be and, as a matter of fact, have been considered either as polyelectrolytes, i.e., polymers with multiple charges of the same sign, or, more frequently (~75% of IDPs), as polyampholytes, i.e., polymers carrying both positive and negative charges [16]. Drawing on a wealth of theoretical studies on polyampholytes [17] and polyelectrolytes [18], IDPs have been modeled as ideal “charge-decorated” polymers and their conformational behavior has been interpreted in the frame of polymer physics [17,19,20,21,22]. Although the paucity of hydrophobic residues weakens the role of “hydrophobic collapse”, i.e., the burial of hydrophobic residues into a protein core, some IDPs can adopt compact conformations [23,24]. As inferred from theoretical studies on polyampholytes, the balancing or unbalancing of opposite charges (i.e., the extent of net charge) is a determinant of IDP conformation, where electrical neutrality enables them to collapse and charge unbalancing results in structural expansion due to repulsive electrostatic forces [25]. In other words, some IDPs can undergo “electrostatic collapse”. Accordingly, the charge content and, most of all, the linear distribution of opposite charges, were shown to be the major determinants of protein compaction and of the amplitudes of conformational fluctuations [15,16,26,27,28]. The scenario is, however, even more complex, as exemplified by recent studies showing that the conformational responsiveness of IDPs to charge clustering is modulated by additional sequence features, such as the proline content [29]. The high conformational heterogeneity that typifies IDPs [30,31,32] prevents their structural characterization by conventional high-resolution approaches and, rather, requires the combined use of multiple, complementary approaches that enable IDPs to be described as conformational ensembles [33,34,35,36,37,38,39].

The peculiar sequence properties of IDPs have been exploited to predict protein disorder and have led to the development of numerous disorder predictors and/or metaservers [40,41,42,43,44]. Several databases gathering information about protein disorder have been developed in the last decade, including DisProt (the largest repository of manually curated intrinsically disordered regions—IDRs, for which disorder has been assessed experimentally) [9,45], MobiDB [46], DisBind [47], FuzDB [48], and Protein Ensemble Database [35,39]. Besides, the information on disorder content has been shown to facilitate the functional annotations of proteins [49,50].

The manifestation of structural disorder in proteins does not obey an “all-or-nothing” rule, with regions of disorder often coexisting with structured regions within proteins. In addition, many “flavors” of disorder exist, thus leading to the concept of “conformational continuum” to include the wide repertoire of documented protein conformations, ranging from fully structured to completely disordered states [30,31,32,51,52]. Furthermore, even in the context of an IDR, the extent of disorder can vary, with IDRs often encompassing transiently populated secondary-structure elements. The latter often serve as Molecular-Recognition Elements (MoREs) or Molecular-Recognition Features (MoRFs), i.e., regions that fold upon binding to a partner [53]. These regions are also known as pre-structured elements (PSEs) [54] or pre-structured motifs (PreSMos) [55,56], which are defined as transient local secondary structural elements present in the unbound state of IDPs.

IDPs undergo variable degrees of disorder-to-order transition upon binding to partner molecules [53,57,58,59]. This phenomenon, known as induced folding or, more precisely, as “folding coupled to binding”, might be regarded as a particular (intermolecular) case of folding. It has been suggested that, in vivo, induced folding limits structural disorder to a transient state, whereby function would arise only once a precise structure is formed as a result of binding to a specific partner [60]. However, some of these “proteins waiting for a partner” (PWPs) [60] have been shown to maintain structural disorder also inside the cell [61,62]. Exploring the conformation and the dynamics of IDPs in their natural intracellular environment is of paramount importance. The viscosity of the cytoplasm, the macro-molecular crowding, post-translational modifications (PTMs) and the presence of a myriad of potential interactors are all factors that can considerably impact on IDP conformation and dynamics. The advent of in-cell spectroscopic techniques (Nuclear Magnetic resonance—NMR—and Förster Resonance Energy Transfer—FRET) has made it possible to start addressing this point. In-cell NMR spectroscopy, a technique that has been pioneered by the groups of Gary Pielak and Philipp Selenko, has attracted much interest, since it enables investigating IDP conformation inside living cells at atomic resolution (for a review see [63]). The group of Ben Shuler has recently demonstrated the feasibility of probing protein dynamics from the ms to the ns regime in live eukaryotic cells, using confocal single-molecule FRET spectroscopy [64]. In addition to NMR and FRET, recent developments in the field of Electron Paramagnetic Resonance (EPR) spectroscopy have made possible the investigation of IDP conformation and dynamics in cellula by the use of a bio-resistant nitroxide spin label [65]. In-vivo crosslinking coupled with mass spectrometry (XL-MS) provides distance constraints useful to model protein conformations and supramolecular complexes [66]. These innovative approaches pave the way towards structural investigation of IDPs inside living cells.

Irrespective of whether they fold or not in the cellular context, PWPs have the distinct properties of not being able to fold in vitro under physiological conditions of pH and salinity. In this respect, they clearly differ from the “normal” globular proteins, which can spontaneously fold under physiological conditions utilizing their “folding code” embedded in their amino acid sequences. The fact that PWPs cannot fold by themselves but need a specific partner indicates that their folding code is incomplete. Thus, PWPs possess peculiar and unique features, which they have been selected for, including their ability to be engaged in folding-upon-binding events [36].

Furthermore, it is worthy to underscore that induced folding is often not complete, leading to so-called “fuzzy” complexes that retain significant residual structural disorder [67]. In particular, after binding to the target, IDPs may either sample a number of conformations at the surface of the partner (i.e., static fuzziness) or preserve fuzzy appendages in the disordered state (i.e., dynamic fuzziness) [48,67,68,69,70]. Protein–protein interactions involving disordered regions (i.e., regions that are disordered in isolation, in their unbound form) are endowed with unique and functionally advantageous properties. The entropic penalty associated with the disorder-to-order transition affects the affinity of the interaction, enabling uncoupled tuning of specificity and affinity ([71] and references therein). The partial pre-configuration of MoREs can, however, attenuate this entropic penalty. As such, IDPs can easily regulate their affinity towards partners by simply modulating the extent to which MoREs/MoRFs/PreSMos/PSEs are pre-configured (i.e., the extent to which they presage the bound conformation). In a similar manner, “fuzziness” offers a way to modulate affinity. Residual disorder in IDP complexes (in the form of fuzzy appendages) provides a way to attenuate the entropic penalty associated to the disorder-to-order transition thereby, ultimately, modulating affinity. Therefore, the involvement of IDRs in protein–protein interactions that need to be finely tuned provides an exquisite means to modulate the interaction strength: by tuning the extent of pre-configuration of the binding motifs and/or the amount of residual fuzziness, an optimal binding strength can be achieved [72].

The abundance of protein disorder fluctuates across proteomes [73,74,75,76]. The fraction of disordered residues does not depend on proteome size and is, rather, tied to organism complexity [77]. Although the notion that structural disorder increases with the complexity of the organism has become widely accepted, the highest levels of disorder are not observed in the most complex metazoan eukaryotes (e.g., in humans), but in single-celled eukaryotes (i.e., protists) that are characterized by a host-changing lifestyle [78]. The prevalence of disorder in either complex organisms or in organisms living in a fast changing environment reflects an increased need for regulation, where IDPs are ideally suited to exert regulatory functions. Structural disorder is known to be a determinant of interactivity [78,79,80]. The lack of a unique 3D structure indeed confers a high plasticity to IDPs and hence enables them to interact with multiple, structurally different partners and to fold differently, a property known as “context-dependent binding” [57,81,82,83,84]. Because of this promiscuity, IDPs occupy a key position in regulatory pathways that typically require multiple, low-affinity interactions. The ability to interact with multiple partners, together with other functional advantages including buffering of deleterious mutations [85], explains the abundance of disorder in viruses [86,87]. Intrinsic disorder represents an ergonomic solution for the virus to encode fewer proteins with more functions, while keeping the genome size as small as possible [87,88].

**Figure 1:**
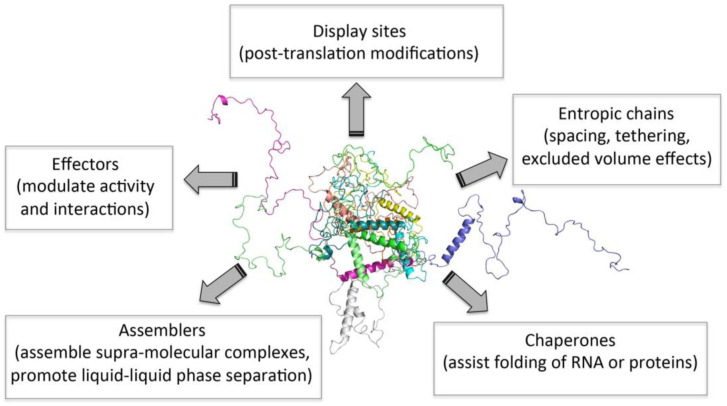
Functions of intrinsically disordered proteins (IDPs). Figure inspired by [48].

It is now clear that structural disorder provides multiple functional advantages, with IDP/IDR functions arising either directly from their disordered nature (i.e., entropic chains) or from molecular recognition (Figure 1). In two decades, intrinsic disorder has, thus, passed from being considered a pure heresy to be regarded as a widespread, functionally relevant biological phenomenon. Beyond being instrumental for regulation and signaling, and for the establishment of complex molecular partnerships, it has been recently recognized that intrinsic disorder also plays a critical role in “liquid–liquid phase separation” (LLPS) or condensation phenomena [15,89,90,91,92].

**Figure 2:**
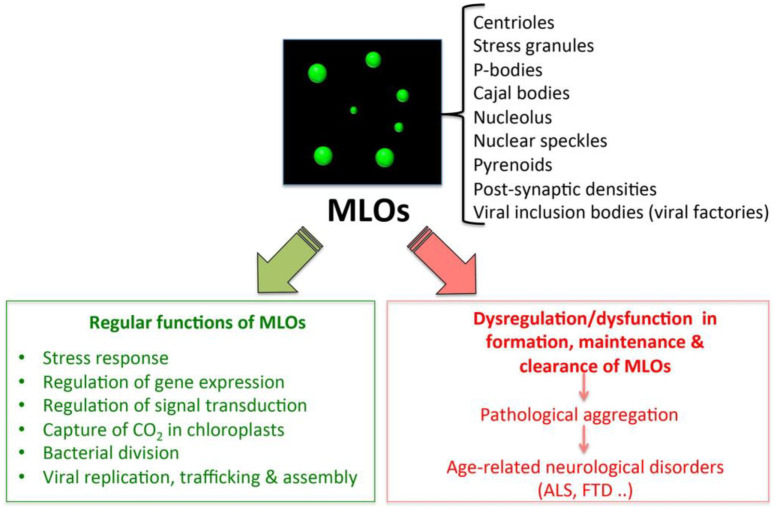
Functions and dysfunctions of liquid–liquid phase separation (LLPS)-driven membrane-less organelles (MLOs).

Intracellular phase-separation and transitions by proteins have attracted much attention in the last decade, in light of the multitude of physiological and pathological processes, in which they are involved [26,90,93,94,95]. In particular, LLPS underlies the formation of the so-called membrane-less organelles (MLOs) (e.g., Cajal bodies, processing bodies (PBs) [96], nucleolus, stress granules (SGs) [97], centrosomes, and aggresomes [98], whose function is essential for the cell and whose dysfunction is associated to various pathologies, including age-related disorders (Figure 2) [26,90,93,94,95].

## 2. Introduction to LLPS

### 2.1. General Concepts of Phase Separation

A physically homogeneous system consists of a single phase and is said condensed when formed by a liquid or a solid. Miscible components can exist in homogeneous solutions or undergo de-mixing, depending on the interactions among solute and solvent molecules. Phase separation can become energetically favored, in spite of the entropic loss entailed by the formation of a two-phase system [99,100,101]. Among possible transformations of a chemically homogeneous system, LLPS is a thermodynamically-driven, reversible phenomenon consisting in de-mixing into two distinct liquid phases, with different solute concentrations [102,103]. The equilibrium between mixing and de-mixing is strongly dependent on the component concentrations, besides temperature, pressure, pH, crowding agents, etc.

**Figure 3:**
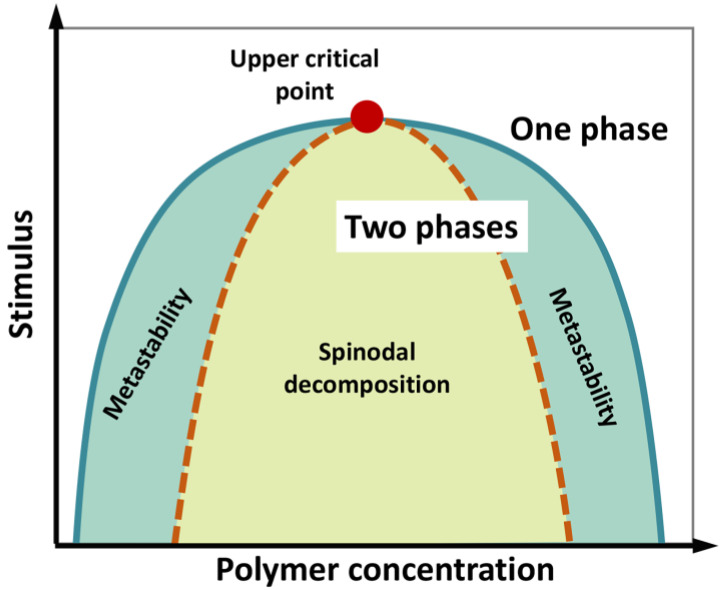
Schematic phase diagram of a colloidal system (e.g., a polymer in water) displaying an upper critical point (filled red point), above which no de-mixing occurs. According to polymer concentration and external stimuli (temperature, pH, ionic strength, etc.), the system consists in a well-mixed, single phase, or two separated phases. The diagram illustrates the coexistence or binodal curve (green) and the spinodal curve (dotted brown). The region in between the binodal and spinodal curves (light-green region) can correspond to metastable, supersaturated solutions. In the region enclosed by the spinodal curve, single-phase mixture is unstable and phase separation (“spinodal decomposition”) spontaneously occurs, in the absence of nucleation, being limited by molecule diffusion. Figure adapted from [107].

We underline that phase separation of a liquid system does not necessarily imply a phase transition (i.e., a state change from liquid to solid), although the latter can take place during the “maturation” of liquid biological condensates. A phase diagram can be constructed by systematically screening, for instance, temperature and concentration, to determine the region in which phase separation occurs (Figure 3). The phase diagram is characterized by a “binodal” (coexistence) curve, which encloses the whole region where phase separation is thermodynamically accessible. A “spinodal” curve encloses an inner region where the single-phase mixture is unstable and phase separation spontaneously occurs, in the absence of nucleation and perturbation, being limited by molecule diffusion and giving rise to two components with similar fractional volume and typical intertwined structures (“spinodal decomposition”). In the region between the two curves, phase separation requires the formation of condensation seeds, through a nucleation step that limits the rate of the de-mixing process. Here, if phase separation does not occur, the lone phase representing the system reaches “super saturation”. This is a “metastable” condition, which can originate a two-phase system even in response to subtle perturbations [104,105,106,107]. 

In the regime of phase separation, liquid droplets can fuse and coalesce, undergoing Ostwald ripening (i.e., larger droplets grow at the expense of smaller ones), and dripping, according to their viscosity and surface tension [108]. A “phase behavior” can be easily figured out for compounds of different chemical nature (hydrophobic/hydrophilic), such as oily substances and water, which are notoriously immiscible [102]. Immiscible substances differ in their partition coefficient (*K*), which is calculated as the ratio of concentrations of a compound in a mixture of two immiscible solvents at equilibrium (typically, octanol and water to obtain *K_ow_*). The greater the difference between *K_ow_* of two molecules, the lower the concentration limit (i.e., solubility). As an example, very small amounts of aniline (*K_ow_* = 1.076 Log L/Kg) are completely miscible in water at any temperature below 441 K. As aniline concentration increases, the two components separate into two liquid phases [109]. Other than temperature and concentration, several factors, including pH, pressure and salts, influence the phase behavior of solution systems.

### 2.2. Phase Separation in Protein Systems

Phase separation has recently emerged as a ubiquitous principle of cellular organization, underlying many biological processes, the list of which is growing rapidly [89,92,95,96,110,111,112,113] (Figure 2). According to the PubMed database, almost 3000 scientific articles have contributed in the last decade to highlight the physiological and pathological relevance of LLPS, which was found to occur in the cell cytoplasm, in the nucleoplasm [88], as well as in vitro for many purified proteins [114]. The growing interest towards LLPS has fueled studies aimed at gathering information on proteins undergoing LLPS in a structured and knowledgeable manner, while providing a wide range of information on the biophysical driving forces, the biological function and the regulation of these systems. These efforts have led to the development of dedicated databases [115], such as PhaSePro (https://phasepro.elte.hu) [116] and LLPSDB (http://bio-comp.org.cn/llpsdb/) [117], two manually curated repositories, based on experimentally verified cases of LLPS.

In analogy with the systems explored by classical physical chemistry, cellular LLPS causes homogeneous solution of macromolecules to separate into two phases. Liquid droplets form, which are enriched in condensed biomolecules and surrounded by a “diluted” phase, depleted of those components [93,99,101]. As long as a genuine liquid nature is conserved, polymer molecules can diffuse within the dense phase and between the dense and diluted phases [101]. It is mesoscopically evident that the de-mixing occurs rapidly and dynamically, in response to concentration changes of some cellular components, or to environmental stimuli, which ultimately result in changes of molecular interaction affinity. Fluid droplets can form/dissolve reversibly as they are held together by a network of weak and transient interactions [103,118,119,120]. Several in-vivo and in-vitro studies suggest that LLPS preferentially involves IDPs/IDRs because of their peculiar conformational properties [84,114,118,121,122,123,124]. Structural studies on the Tau protein suggest that phase separation is driven by its conformational expansion, coupled with solvation and large-scale fluctuations, while transient, non-covalent intermolecular contacts maintain the internal fluidity [125]. Nonetheless, LLPS has also been observed for ordered, globular proteins. Bovine serum albumin was used as a model to experimentally describe the kinetics of temperature-driven de-mixing [126]. Overall, condensate immiscibility appears as a very general feature in biological systems, which could also be exploited to create synthetic membrane-less compartments [127,128]. Theoretical and computational studies on protein de-mixing [127,129,130,131] confirm that protein phase diagrams are mainly shaped by salt concentration and solvent-mediated interactions, in turn influenced by temperature, their effects being cumulatively translated into “interfacial tension”, i.e., the work required to increase the size of the interface between two adjacent, immiscible phases. [127]. Sequence-specific properties, such as the presence of charged and aromatic residues, may then stabilize the de-mixing arrangements [130]. The role of salts on protein de-mixing has been considered for the peculiar case of multivalent ions [132,133]. It has emerged that some multivalent cations (e.g., yttrium) bind to proteins by an entropically-driven process, due to changes in the structure of the hydration shell. Once the protein–cation complex is formed, the multivalent ions can act as bridging elements to drive intermolecular interactions [132,133]. 

Networking is a very relevant property in LLPS, mainly related to the modularity and the “multivalence” of the involved macromolecules. Multivalence refers to the presence of multiple, repetitive interaction sites within a polypeptide or nucleic acid [134,135]. Again, IDPs and IDRs, with their low-complexity domains (LCDs) and multiple interacting sites (i.e., short linear motifs, SLiMs) [136,137,138], have a privileged role among cellular components undergoing LLPS [134,136], causing the formation of densely packed liquid phases [139] often undergoing “gelation” when the network of interactions span the entire system, i.e., the whole droplet [140]. In this context, multivalent proteins have also been described as a peculiar type of “associative polymers” endowed with “stickers-and-spacers architecture”, where the stickers are motifs and interaction sites, and the linkers are those protein regions contributing to sticker networking [141]. 

The “molecular grammar” of interactions governing LLPS is now beginning to be understood, although it is still in its infancy [142]. IDPs that drive LLPS typically display low-complex sequences, characterized by long stretches with overall low diversity of amino acids [78]. The sequences are often repetitive and are enriched in glycine, polar sidechains (glutamine, asparagine and serine), positively charged sidechains (Arg and Lys), negatively charged sidechains (Asp and Glu), and aromatic sidechains (Phe and Tyr). The sequences of interest often encompass multiple short motifs such as YG/S-, FG-, RG-, GY-, KSPEA-, SY- and Q/N-rich regions, and blocks of alternating charges [143]. Interactions occur via different residue types, such as Phe and Tyr involved in π-π stacking and π-cationic interactions with Lys and Arg residues, polar and charged residues [139,142,144,145,146]. Distribution of charged amino acids along the sequence has also been suggested to have a role in the coacervation propensity [147], with electrostatic interactions between blocks of oppositely charged residues being associated to stronger driving forces—through long-range, interchain attractions [143] and larger coacervation windows [129] than sequences with interspersed charges. This is in agreement with the general envision that phase behaviour of multivalent proteins is governed by patterning rules of “sticker” motifs of different nature [148]. The compositional rules emerging from in-vitro and in-cell studies also suggest the existence of two kinds of components, with distinct roles: multivalent proteins forming scaffolds and low-valence, client molecules tethered onto those scaffolds. The state of a droplet can change rapidly, through changes in the composition and concentration of scaffold proteins or through changes in their valence [149].

### 2.3. Techniques to Study LLPS

LLPS can be documented and monitored using various methods of varying complexity. These include simple macroscopic observation of purified samples and assessment of their turbidity, measurements of optical density and light scattering, light microscopy (fluorescence-based or contrast-based) and super-resolution microscopy studies. LLPS can be experimentally characterized both in vitro and *in vivo*. For in-vitro assays, the minimal components are combined in buffers to recapitulate LLPS, whereas in-vivo studies are carried out in cells, tissues or even animals. Here we provide a concise description of the most commonly used methods. A more exhaustive description of the available methods can be found in the excellent reviews by Alberti et al. and Mitrea et al. [108,150].

For in-vitro studies, a very simple clue of LLPS is the appearance of sample turbidity. Phase separated samples become turbid because the droplets (i.e., the coacervates) scatter light. They can be detected by optical density measurements (typically at wavelengths of 340, 400 or 600 nm) or by direct static light scattering. The latter approach allows determination of the onset of scattering of a dilution series, thus enabling identification of the threshold concentration for droplet formation, *C_sat_*. 

One caveat of simple turbidity measurements resides in the fact that they detect a variety of assemblies and do not differentiate their shape, size or the mechanism underlying their formation. Turbidity measurements should thus be used in conjunction with microscopy. Under a microscope, the separation process is materialized by the appearance of randomly moving, spherical and dynamic droplets, of micrometric size. Coacervates are to be distinguished from aggregates that are often irreversible and irregularly shaped. Quantitative image analysis enables assessing coacervate sphericity, deformability under shear stress and coalescence abilities, three properties commonly regarded as reflecting the liquid nature of the bio-condensates. One caveat is that it may be impossible to detect diffraction-limited assemblies with simple methods, and super-resolution microscopy, which combines optical inputs with mathematical analysis to construct images of specimens with resolution from 2- to 10-fold below the diffraction limit, may be required. 

An easy way to demonstrate in vitro that an assembly forms through LLPS is to define a saturation concentration C*_sat_*, where for C < *C_sat_* the protein is diffuse in solution, and for C > *C_sat_* dense droplets can be observed. The volume fraction of these dense droplets increases when the concentration is increased further, while the concentration in the light phase (*C_L_*) remains constant. This behavior is typical of phase separation and constitutes a strong support for LLPS. The increase in the volume fraction of the dense phase (*C_D_*) can be estimated via light scattering or centrifugation. The use of fluorescently labeled components allows estimation of the concentration inside *C_D_*, provided that standard curves for the fluorophore at different concentrations are obtained under identical imaging conditions.

Centrifugation of systems in the two-phase regime, and the ensuing separation of light and dense phases, allows precise measurements of the concentrations of two coexisting phases, *C_L_* and *C_D_*, as a function of a given physico-chemical parameter. LLPS of the stock solution is induced by changing the solution conditions, and after incubation, the dense phase is sedimented by centrifugation [151]. An aliquot of the light phase is removed, and its concentration determined spectroscopically (e.g., by measuring the absorbance at 280 nm or fluorescence intensity if dealing with a fluorophore-labeled protein). This experimental design enables assessing whether *C_L_* remains constant when using different protein concentrations [123,152]. It should be emphasized that processes such as fibrillation may interfere with LLPS, making accurate determination of *C_L_* difficult. Thus, microscopic analysis that can distinguish liquid droplets from aggregates or fibers is, once again, recommended. 

Fluorescence Recovery After Photobleaching (FRAP) is commonly regarded as a technique for ascertaining the liquid versus solid nature of biomolecular condensates. FRAP monitors the diffusion of fluorescent molecules within a photobleached region and is used to assess macromolecular mobility within phase-separated condensates both in vitro and in vivo. A specific region of interest is illuminated by a high-intensity laser, at the excitation wavelength of the fluorophore, to irreversibly convert the molecules to a dark state. The diffusion of fluorescent molecules from outside to inside this region is then quantified by measuring the variation in fluorescence intensity as a function of time. From the evolution of the fluorescence intensity within the region of interest, the rate of (or half-time for) fluorescence recovery of a photobleached component, and the extent of fluorescence recovery (referred to as the mobile fraction), can be derived. Fast exchange rates (i.e., in the second range) characterize liquid-like assemblies. If the droplet is large enough, bleaching within droplets allows probing intra-droplet dynamics [110,153]. Importantly, FRAP can also be useful in assessing whether the droplets are spatially homogeneous based on the pattern of recovery. 

Many proteins, either in the presence or absence of RNA, are capable of undergoing LLPS in vitro when a sufficiently high concentration is reached. Therefore, the mere observation that a given protein undergoes LLPS in vitro does not imply per se a functional relevance, and proving LLPS occurrence in vivo is, therefore, of paramount importance. Demonstrating that an assembly results from LLPS in the cellular context is challenging and much caution should be taken before drawing definite conclusions, especially when interpreting over-expression data. An ideal approach would be to attempt at establishing phase diagrams in living cells [119]. To this end, it is necessary to vary in a controlled manner the expression levels of the protein of interest. The measured *C_sat_* can then be directly compared to the cellular concentration of the protein under a given condition, enabling to conclude whether phase separation can take place or not. 

Currently, the commonly accepted criteria for considering that an assembly is a phase-separated structure are (i) spherical shape, (ii) ability to fuse (coalescence), (iii) deformability under shear stress and (iv) fast recovery from photobleaching. However, it should be kept in mind that fast recovery rates from FRAP can arise from multiple reasons and several potential sources of artifacts may affect the quantitative interpretation of photobleaching results (reviewed in [108,150]). Thus, none of the above features is sufficient on its own to unequivocally demonstrate that a structure is formed via LLPS.

### 2.4. Functional and Dysfunctional Aspects of LLPS 

The structural and material properties of LLPS condensates resulting from LLPS are intimately linked to their function as reaction crucible and organizational hub [99]. For instance, signaling complexes trapped inside condensates can better coordinate cell response to environmental stimuli [154]. Overall, sequestration inside LLPS droplets, because of the high viscosity, can exclude some interactions (with “external” molecules) and increase some others, by enhancing the probability and kinetics of molecular interactions and enzymatic reactions among “internal” molecules [155,156,157]. 

The “maturation” of liquid droplets toward gel or solid state has also been reported [92,102,108,140,158]. Amyloid-like fibers can form hydrogels, which are non-dynamic and rather stable, as they dissolve only under harsh conditions (i.e., high salt or denaturant) [159,160,161]. Liquid-to-solid transitions have been proven functional or dysfunctional/pathological for several different proteins. One example of functional liquid-to-solid transitions is offered by SGs, formed upon heat stress by the yeast protein Pab1. This protein forms glassy droplets that play an adaptative role, promoting cell survival upon heat shock [108,162]. Dysfunctionally persistent SGs were obtained from the RNA-binding protein hnRNPA1, whose fibrillation is enhanced in protein-rich droplets [121]. Mutations in fused-in-sarcoma RNA-binding protein (FUS) and in heterogeneous nuclear ribonucleoprotein A1 (hnRNPA1) are involved in familial forms of amyotrophic lateral sclerosis (ALS) and frontotemporal dementia (FTD) [163,164,165]. Both proteins form liquid droplets at moderate concentrations, but these droplets can nucleate amyloid-like fibers and begin to jellify over time, a process accelerated when the protein exhibits disease-associated mutations [119,121,166]. IDRs involved in LLPS are therefore implicated in neurodegeneration and able to undergo fibrillation [119,121,152,160,167]. Consistently, many protein domains that promote phase separation have been described as prion-like [168].

The liquid-droplet environment also strongly accelerates fibrillation of TAR DNA-binding protein of 43 kDa (TDP-43). Rapid assembly of fibrillar aggregates involves the low complexity domain of TDP-43 and develops from mature liquid droplets [169]. Overall, one can assume that the occurrence of LLPS is prodromal to a wide range of possible transitions, giving rise to a continuum of material properties, according to the nature of involved proteins and the mechanisms of their interactions [99,170]. 

The rapid assembly and disassembly of liquid condensates compared to membrane-delimited organelles enables a rapid cellular reorganization and hence a rapid response to stimuli. Because IDPs/IDRs are known to be heavily targeted by PTMs and alternative splicing, and because both kinds of events can rewire interactomes, the involvement of IDPs/IDRs in LLPS offers an exquisite manner to regulate the spatio-temporal formation of these cellular condensates [154,171]. 

Considering that viral proteins are enriched in IDRs and are multivalent, their ability to undergo LLPS is not surprising, as well illustrated by recent studies that have provided experimental evidence for this phenomenon and have elaborated on its functional implications.

## 3. LLPS by Viral IDPs/IDRs

LLPS by viral proteins has been implicated in a wide array of different steps and regulatory processes in viral replication cycles. A functionally important manifestation of LLPS driven by viral proteins is the formation of so-called “viral factories” or “viral inclusions”. Viral factories are sites where viral replication and assembly take place and where specific viral and cellular proteins, as well as nucleic acids, concentrate. They serve as platforms for optimized viral replication, thanks to selective uptake or exclusion of components and shielding from the host immune defense. Viral factories can be either membrane-delimited or devoid of membranes. In the latter case, they are referred to as “membrane-less replication compartments”. An additional functional manifestation of LLPS by viral proteins is the formation of condensates that are not primary sites of viral RNA synthesis and are, rather, associated with virus assembly and trafficking of viral components (i.e., proteins and nucleic acids). 

A functionally distinct category includes LLPS events that are not associated to viral replication and assembly, and rather interfere with host cell functions. This interference may rely on alteration of host gene transcription or interaction with cellular proteins. We refer to this less well-known phenomenon as “LLPS-mediated interference with host cell functions”. 

Most of the examples of LLPS by viral proteins documented so far concerns membrane-less replication compartments. We anticipate, however, that many additional examples of viral protein aggregation, not primarily recognized as manifestations of LLPS will be in the next future revisited in light of LLPS.

### 3.1. Examples of Viral Proteins Undergoing LLPS Associated with Viral Replication, Assembly and Traffiking

The first report demonstrating LLPS by viral proteins in-vivo concerns the rabies virus (RABV) [172]. A distinctive feature of neurons infected by RABV is the presence of cytoplasmic inclusions known as Negri bodies (NBs). In the case of RABV, as well as of vesicular stomatitis virus (VSV, another rhabdovirus) [173], Ebola virus (EBOV, a filovirus) [174] and respiratory syncytial virus (RSV) (a pneumovirus) [175], these inclusion bodies (IBs) contain all the ribonucleoparticle (RNP) components (e.g., nucleoprotein, N; Large protein, L and phosphoprotein, P) and viral RNAs (genomic, antigenomic, and messengers), which are synthesized inside the particles. Therefore, these IBs can be considered as bona fide viral factories. In 2017, Nikolic et al. showed that RABV NBs are endowed with distinct features of liquid organelles. The authors first analyzed infected cells (fixed and permealized at different times post-infection) using immunofluorescence (with an anti-N antibody), and the cytoplasmic inclusions, found to be devoid of membrane, were counted and classified based on their size. Their location was analyzed by confocal microscopy and then confirmed by electron microscopy on fixed samples. Subsequently, to shed light onto the dynamics of NBs, the authors used live cell imaging of cells infected with a recombinant RABV encoding the P protein C-terminally fused to the mCherry fluorescent protein. From the analysis of the axial ratio of the inclusions, the authors concluded that they were spherical, thus suggesting a liquid-like nature. In agreement, NBs were found to be endowed with capability to fuse with each other (i.e., coalescence), sensitivity to osmotic shock [118] and rapid internal diffusion, as assessed by FRAP [172].

FRAP experiments revealed that the P protein, although more concentrated in NBs, can shuttle between the cytosol and the NBs, thereby possibly enabling recruitment of P cellular partners such as the focal adhesion kinase (FAK) and the major inducible heat shock protein 70 kDa (HSP70), two host proteins with proviral activities [173,176]. Therefore, LLPS is an efficient process to enrich the viral factories in components that are required for viral transcription or replication. On the other hand, LLPS may also exclude proteins with antiviral properties. Indeed, it has been shown that SGs, which contain cell receptors acting as sensors of RNA virus replication [177,178,179], do not fuse with NBs [172].

A minimal NB-like system forms upon expression of the RABV N and P proteins alone, which recapitulate the NB features, indicating a driving role of these viral proteins in LLPS [172]. These proteins are characterized by a high degree of structural disorder and multivalence, as typical of protein components of liquid organelles. In particular, the dimerization domain and the second disordered domain of P (IDD2) have proved indispensable for in-vivo phase separation [172].

RABV RNPs are ejected from NBs by a microtubule-independent mechanism and are then transported away by the microtubule network. The newly formed RNPs can either be incorporated into new virions, upon budding at the cell membrane, or can seed formation of new NBs by secondary LLPS processes. By reconsidering the available literature evidence on spherical structures that form inside infected cells, Nikolic et al. proposed that the formation of viral factories by LLPS driven by viral proteins is a common trait among viruses of the *Mononegavirales* order (i.e., viruses with a non-segmented RNA genome of negative polarity) [172]. Indeed, using similar approaches, this property has been soon after demonstrated for two other *Mononegavirales* members, namely the vescicular stomatitis virus (VSV) [180] and Measles virus (MeV, a paramyxovirus) [181]. In the latter case, maturation of viral inclusions from a liquid-like to a gel-like state, as inferred from FRAP experiments, has been interpreted as a possible regulatory mechanism controlling organelle dynamics to optimize the viral replication cycle. 

Co-expression of N and P proteins from paramyxoviruses also leads to the appearance in transfected cells of spherical inclusions that recapitulate the liquid properties of IBs. In all cases, the protein regions required for IBs formation are intrinsically disordered (for reviews see [182] and [183]). In the case of Borna disease virus (BDV, another member of the *Mononegavirales* order), the P protein alone is sufficient to sustain the formation of IBs with a probable liquid nature [184]. Likewise, the N protein from EBOV is sufficient for IB generation [185]. By contrast, in the case of VSV, co-expression of the L protein is required [180]. While, in the case of RABV and MeV, the N-P inclusions formed in this minimal system have the same liquid characteristics as the viral factories [172,181,186], the liquid-like nature of the spherical inclusions formed by either BDV P or EBOV N remains to be rigorously demonstrated.

**Figure 4:**
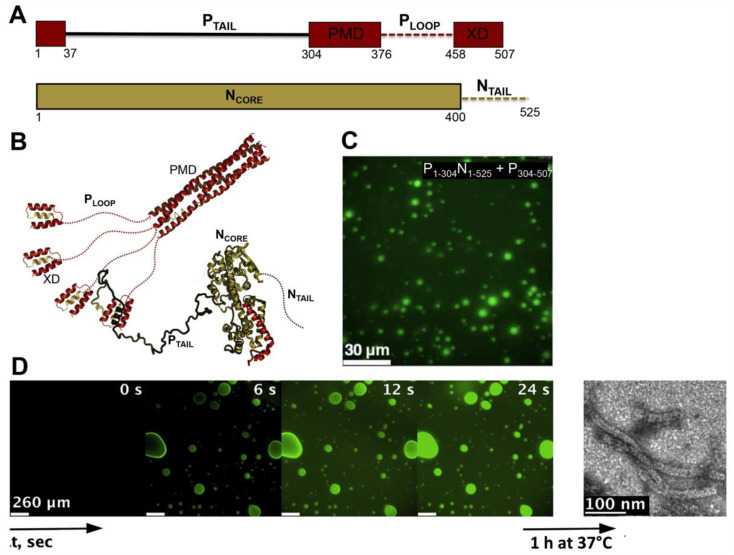
MeV N and P proteins form membrane-less organelles (MLOs) that colocalize with RNA and promote assembly of nucleocapsid-like particles. (**A**) Modular organization of the P and N proteins, where structured regions are shown as rectangles and disordered regions as dashed lines. The N-terminal module encompassing residues 1 to 37 corresponds to the Molecular Recognition element (MoRE) that binds to the monomeric form of the N protein while adopting a kinked α-helical conformation. PMD: P multimerization domain responsible for P tetramerization; XD, X domain. (**B**) Cartoon representation of the P region encompassing residues 304–507. Disordered regions are shown as dotted lines (modified from [183]). (**C**) Fluorescence microscopy image of a mixture containing fluorescein-labeled P_1_-_304_N_1_-_525_ and P_304–507_ where liquid-liquid phase separation (LLPS) occurs. (**D**) Fluorescence microscopy image showing fluorescently labeled RNA diffusing into droplets preformed by mixing P_1−50_N_1−525_ and P_304−507_. RNA colocalizes to N:P droplets and forms nucleocapsid-like particles, as observed by negative-staining electron microscopy after 1 h of incubation at 37 °C. Panels C and D reproduced with permission from [186].

MLOs consisting of MeV N and P proteins also form in vitro upon mixing various purified P constructs with so-called N^0^P complexes (i.e., complexes between the RNA-free, monomeric form of the N protein and the N-terminal disordered region of the P protein) (Figure 4) [186]. Fluorescence and differential interference contrast (DIC) microscopy at various combinations of the different N and P domains, have indicated that the P tetramerization domain, the disordered P_LOOP_ region and the three-helix bundle X domain (XD) are essential for droplet formation (Figure 4A–C). Full-length N, comprising the disordered N_TAIL_ domain, was found to be essential for LLPS to take place (Figure 4A–C) [186]. These results mirror those obtained in vivo with MeV [181] and RABV [172]. 

The combination of P_1−50_N_1−525_ with P_304−507_ was identified as the minimal phase-separating system, capable of forming spherical bi-molecular condensates as measured by fluorescence microscopy and as inferred from increased turbidity [186]. The LLPS phase diagram was established on the basis of turbidity (where an absorbance value higher than 0.1 was interpreted as a sign of phase separation), and led to the identification of threshold concentrations of 10 μM for both proteins, although fluorescence microscopy and negative-stain electron microscopy revealed phase separation at lower protein concentrations. The liquid-like behavior of droplets was inferred by DIC microscopy via observation of fusion events and generation of spherical droplets of increased volume, and further confirmed by FRAP studies of fluorescein-labeled P_1−50_N_1−525_ showing recovery within seconds [186]. Fluorescence recovery of the P protein was found to be considerably slower, a finding that the authors interpreted as reflecting a possible role for the P tetramer as a dynamic scaffold for the liquid droplets. Using time-resolved fluorescence anisotropy, the authors investigated the rotational diffusion of the proteins within droplets and in the dispersed phase. The rotational correlation time of P_1−50_N_1−525_ in the presence of P_304−507_ was significantly reduced compared to P_1-50_N_1-525_ alone, with this difference being observed only under conditions in which LLPS occurs. Similar results were obtained in mirror experiments where the rotational correlation time of P_304–507_ alone or in the presence of P_1−50_N_1−525_ was determined. Although the rotational dynamics is slowed down, both proteins remain liquid in the condensed phase [186]. 

Fluorescence microscopy studies with fluorescently labeled RNA showed that the RNA colocalizes within preformed droplets made of P_1−50_N_1−525_ and P_304−507_, and negative-stain electron microscopy studies (Figure 4D) showed that this colocalization is accompanied by the formation of nucleocapsid-like particles that result from encapsidation of RNA by N protomers [186]. Using NMR spectroscopy, and through comparison with previous studies describing nucleocapsid assembly under non-separating conditions [187], the authors showed that the rate of encapsidation within droplets is enhanced compared to the dilute phase. The finding that LLPS by MeV N and P proteins in vitro correlates with increased nucleocapsid formation, together with the observation that MeV forms liquid-like IBs in infected cells [181], provides strong indication that the formation of droplets is functionally coupled with virus replication. Similar N- and P-containing IBs, often corresponding to sites of active viral replication, were observed in a number of paramyxoviruses, such as human parainfluenza virus 3 (hPIV3) [188], parainfluenza virus 5 (PIV5) [189], mumps virus (MuV) [189], Nipah virus (NiV) [190], and simian virus 5 (SV5) [191]. However, they were not explicitly recognized as resulting from LLPS, in spite of their ability to coalesce and/or their spherical nature, two properties that strongly support the liquid-like nature of these cytoplasmic inclusions. Similarly, IBs formed by N, P and L proteins from human RSV [175] and human metapneumo virus (hMPV, another pneumovirus within the *Mononegavirales* order) [192], have a suspected liquid-like nature, although they were not discussed in light of LLPS. The N and P proteins of all these viruses display the common property of bearing IDRs, further emphasizing the link between intrinsic disorder and LLPS. 

LLPS by viral proteins is not a unique feature of *Mononegavirales* members, and is not restricted to the formation of replication compartments. The eight-partite genome of the influenza A virus (IAV) assembles inside viral inclusions displaying liquid-like properties and containing the host protein Rab11, a marker of recycling endosomes, as assessed by immunofluorescence, transfection by fluorescent tagged proteins, FRAP and time-lapse imaging [193]. Electron microscopy and confocal imaging show that the formation of these liquid organelles is spatially regulated, being detectable at the endoplasmic reticulum (ER) exit sites and dependent on vesicle trafficking between ER and Golgi. This is an interesting example of crosstalk between MLOs formed by LLPS and classical intracellular organelles [156,194]. The cytosolic puncta (i.e., the small, spherical condensates) form and dissolve reversibly and react promptly to changes in the chemical environment, such as hypotonic stress and addition of 1,6-hexanediol, a reagent known to perturb LLPS [185]. The viral inclusions can form even when only one type of RNP is expressed, thus revealing that their formation does not depend on RNA–RNA interactions among distinct ribonucleoproteins, as previously thought. Again, a link between intrinsic disorder and the liquid inclusions formed during IAV infection can be established: the nucleoprotein found in the viral inclusions contains an IDR that regulates viral genome packaging through interaction with both viral RNA and the plasma membrane [195].

The tegument protein UL11 from Herpes simplex virus 1 (HSV-1) has been shown to undergo LLPS in vitro, based on macroscopic and time-lapse imaging by a stereo light microscope [196]. The protein is endowed with a high degree of structural disorder and multivalent interaction properties. Structural disorder seems to be a conserved feature within the family of proteins homologous to UL11, advocating for a functional relevance [196]. UL11 interactions with the viral protein UL16 are thought to mediate the secondary envelopment process of newly formed capsids by bridging outer and inner tegument layers, where UL11 and UL16 respectively reside. UL11 also interacts with UL21, glycoprotein E and RNA. It is likely that its capability to undergo LLPS in vitro reflects a physiological role of phase separation in tegument assembly and final stages of virus maturation.

Another example refers to the human immunodeficiency virus 1 (HIV-1) nucleocapsid protein (NC), a cleavage product generated from pr55Gag that controls several steps of retroviral replication, such as selective RNA packaging and Gag oligomerization [197]. NC contains two Zn fingers (ZnF), which have been already recognized as ideal therapeutic targets [198]. In that study, the authors show that NC undergoes Zn^2+^-dependent LLPS, producing coacervates with liquid-like features in vitro and inside cultured cells, by DIC and time-lapse fluorescence confocal microscopy, either in isolation or within the full-length Gag protein. Interestingly, variants bearing mutations in the conserved ZnF motifs cannot undergo LLPS. Zn^2+^ chelation results in reduced colocalization of NC and viral RNA, as well as altered NC and viral RNA trafficking between the nucleus and the cytoplasm, suggesting that Zn^2+^-loaded NC may promote RNP formation and nuclear export for virus production. This is an illustrative example of a crosstalk between LLPS triggered by a viral protein and host cell MLOs. The result is relocalization of viral RNA to SGs, along with increased Gag expression and reduced viral production.

Again, a role for structural disorder and protein multivalence has been hypothesized, since the NC ZnF overlaps with a prion-like domain (PLD), which is conserved in other retroviral Gag proteins capable of undergoing LLPS [199]. As already mentioned above, evidence of PLD-driven LLPS has long been recognized in proteins involved in neurodegeneration, with a possible mechanistic link between coacervate formation and amyloid fibrillation [121]. The study by Monette et al. [197] extends the spectrum of physio-pathological roles of PLD-driven LLPS to Zn-dependent RNA trafficking and crosstalk between viral and cellular LLPS transitions.

Finally, SARS-CoV-2 proteins have been involved in LLPS of potential functional relevance. The N protein of this virus undergoes phase separation in vitro in a salt-, pH- and RNA-dependent manner, as assessed by DIC and fluorescence confocal microscopy [200]. It is hypothesized that this behavior could play a role in vivo by promoting nucleocapsid formation in a manner reminiscent of *Mononegavirales* IBs. Moreover, the N protein not only drives LLPS but also partitions as a client protein [201] into liquid phases formed by the host proteins hnRNPA2, FUS, and TDP-43, suggesting that the N protein can recruit host SG components to facilitate virus replication.

### 3.2. Examples of LLPS-Mediated Viral Interference with Functions of Animal Host Cells

Two major mechanisms of LLPS-mediated viral interference with animal host cells can be distinguished: one based on direct interaction of viral condensates with specific host genes, and one based on interaction with cellular proteins. These latter can lead to sequestration of key cell proteins, such as those triggering the cell innate immune response, or to exclusion (or sequestration in an inactive form) of cell sensors detecting pathogen associated molecular patterns (PAMPs).

Epstein–Barr virus (EBV, a DNA virus) provides an example of the former type of interference, which targets the host genes. Its transcription regulators EBNA2 and EBNALP form liquid condensates in the nuclei of infected cells [202]. These puncta display an ATP-dependent FRAP behavior. EBNA2 and EBNALP coacervation in vivo is associated with enhanced expression of target genes, like MYC and Runx3, and their expression is suppressed by chemicals that perturb LLPS, such as 1,6-hexanediol. Furthermore, EBNA2 nuclear puncta colocalize with MYC and Runx3 superenhancer locus of DNA, as assessed by in-situ immunofluorescence assays [202]. These results show that EBNA2 and EBNALP nuclear condensates drive superenhancer-mediated transcription of target genes and provide evidence of the diverse physiological roles that LLPS can play in viral life cycle. The molecular determinants of EBNA2 and EBNALP condensation have been investigated, as well. Predicted IDRs from these proteins are sufficient to drive the LLPS process in vivo and in vitro, in line with a key role of structural disorder in driving phase separation. The amino acid composition of these IDRs is strongly biased, being enriched in Pro, Gly, and Arg residues. Alanine mutagenesis reveals a specific role of Pro in mediating the transition, while Gly and Arg can be mutated without heavily affecting puncta formation. The in-vitro phase separation process is triggered by the crowding agent PEG 10,000 and suppressed by NaCl above 50 mM.

As already mentioned, we suspect that the few studies that to date have reported the formation of fibrillar aggregates by viral proteins might be revisited in light of LLPS, where those fibrils might correspond to solid-like inclusions formed upon the maturation of liquid-like condensates. From those few reports, the formation of fibrillar aggregates by viral proteins was shown to have various effects, including (*i*) blockade of key cellular processes, such as necroptosis, via the formation of hybrid amyloids of viral and host origin [203], (*ii*) interference with the integrated stress response (i.e., blockade of SG assembly by Ebola virus VP35) [204] and (*iii*) suppression of host cell RNA synthesis through the sequestering of host transcription factors (i.e., NSs protein from Rift Valley fever virus, RVFV) [205]. In this latter case, the amyloid-like nature of the aggregates has been proven through Thioflavin S staining, super-resolution microscopy and quantitative live cell imaging of infected cells. Using mutated viruses encoding a non-amyloidogenic form of the NSs protein, a functional link was established between the formation of such fibrils and virulence, and the underlying mechanism was shown to rely on suppression of interferon (IFN) responses (e.g., reduction in IFN-β expression and degradation of PKR) [206]. All these effects would provide examples of LLPS-mediated viral interference with host cell functions through interaction with cellular proteins.

In the same vein, the V protein from Hendra virus (HeV, a paramyxovirus) was shown to undergo a liquid-to-gel phase transition in vitro. The minimal V region responsible for this property, referred to as PNT3, has been identified within the intrinsically disordered N-terminal domain of V [207]. PNT3 was found to form amyloid-like fibrils both in the liquid and hydrogel state. Fibrils not only form in vitro, but also in the cellular context, as they are observed in human embryonic kidney (HEK) cells after the isolated expression of PNT3. In light of the role that the V protein plays in the evasion of the antiviral type I IFN-mediated response [208,209,210], in preventing the detection of viral RNA by the innate immune sensor MDA5 [211], in inhibiting the production of chemokines in vitro and in modulating the inflammatory response in vivo [212], it is conceivable that the amyloid-like structures formed by the HeV V protein may sequester key cell proteins involved in the host innate immune and inflammatory response, thereby contributing to the high pathogenicity of this virus.

### 3.3. Examples of Potential Interference with PLANT LLPS and MLOs by Phytoviruses 

Although the majority of studies on the roles of LLPS in viral translation and replication have been described in mammalian systems, the evidence linking plant virus infection to various MLOs is also starting to accumulate (reviewed in [213]), although a clear connection with structural disorder has not been highlighted as yet. For example, it has been shown that some proteins of phytoviruses can localize to plant MLOs, as illustrated by the capsid protein (CP) from tomato yellow leaf curl virus (TYLCV, a member of the *Geminiviridae* family), which was found in the nucleolus and in some cytoplasmic speckles of the host plants *Nicotiana benthamiana* and tomato [214]. It was also pointed out that, in analogy with animal viruses, replication and translation of plant viruses require RNA-containing granules such as SGs, where translationally inactive host mRNAs are stored, as well as processing bodies (PBs), where mRNA decay may occur [213]. 

Although information about utilization of LLPS and MLOs by plant viruses remains very limited, there are some important hints indicating that these viruses highjack cellular phase-separation mechanisms, as well. For example, plants (e.g., *Arabidopsis thaliana*) contain a homologue of the animal Ras-GAP SH3 domain-binding protein (G3BP), a protein known to oligomerize in response to stress and to nucleate SGs [215], to interact with the conserved short linear motif Phe-Gly-Asp-Phe (FGDF-type motif) of viral proteins (e.g., nsP3 proteins of Semliki Forest virus and Chikungunya alphaviruses) [216,217,218,219], and to be a potential component of viral factories and replication complexes of mammalian viruses, such as poxviruses, hepatitis C virus, and vaccinia virus [220]. Since plant SGs contain G3BP-like proteins, and FGDF-type motifs are found in proteases of closteroviruses, potyviruses, and waikaviruses, it is likely that plant viruses can interfere with formation of SGs via interactions with G3BP, as well [213]. 

Another plant homologue of the animal SG-specific protein is the oligo uridylate binding-protein 1 (UBP1), a protein that facilitates the nuclear maturation of plant pre-mRNAs [221]. UBP1 possesses several RNA-binding domains and a PLD needed for SG biogenesis. In *N. benthamiana*, this protein is a component of RNA granules induced by potato virus A (PVA; genus Potyvirus) [214], suggesting that it can be highjacked by the phytovirus for its translation. The accumulation of UBP1 in virus-induced RNA granules provides an example of how a virus controls host LLPS by targeting a host IDR involved in SG formation. Silencing the PVA suppressor protein HCPro (helper component proteinase) was shown to initiate formation of RNP granules containing viral RNA, ribosomal protein P0 and several PB and SG markers [222].

### 3.4. LLPS in Prokaryotic Cells and Interference by Bacteriophages: A Hypothesis

The preceding sections have discussed the roles of viral proteins in interfering with LLPS and MLO biogenesis in animals and plants. However, viruses can also infect bacteria, and it is likely that bacteriophages could exploit some bacterial-specific LLPS, as well. This hypothesis is based on the fact that phase separation is known to drive numerous cellular processes in bacteria, as well, and there are several bacterial-specific MLOs. However, studies on the abundance and variability of bacterial MLOs and on the prevalence of LLPS for bacterial proteins in vivo is falling behind the analogous studies on eukaryotic cells, mostly because of the small size of prokaryotic cells and associated challenges of studying structures near or below the diffraction limit. Despite these issues, recent years have witnessed a rise of reports dedicated to bacterial LLPS and MLOs, clearly indicating that bacteria indeed contain biomolecular condensates [223]. Among these, there are ”*bacterial microcompartments*” (BMCs), which have been described as proteinaceous, 40–200 nanometers organelles of icosahedral or quasi-icosahedral shape [224,225] that encapsulate enzymes, colocalizing them with the cognate substrates and cofactors [226,227,228]. The discovery of BMCs in an ever-increasing number of heterotrophic bacteria, and their involvement in the utilization of special carbon and energy sources has suggested an evolutionary role in metabolic innovation, to cope with changing environments [226,227,228]. Choline degradation BMC found in the uropathogen *Escherichia coli* 536 represents an example of pathological relevance [224]. Moreover, due to their prominent solid-like, semi-crystalline nature, a clear link between LLPS and BMCs has not been established yet. Likewise, the underlying mechanism leading to the peculiar scaffold of these biomolecular condensates has not been investigated in depth.

Another example of prokaryotic condensate is that offered by the ABC transporter Rv1747 from *Mycobacterium tuberculosis* and its homologue from *Mycobacterium smegmatis*. These proteins are capable of forming liquid-like dynamic *foci* and two-dimensional nanoclusters at the cell membrane [229]. Both proteins contain a cytoplasmic regulatory module consisting of two phosphothreonine-binding Forkhead-associated domains, joined by an intrinsically disordered linker with multiple phospho-acceptor threonine residues. In the bacterium *Caulobacter crescentus*, LLPS leads to the formation of bacterial RNP-bodies (BR-bodies) in the cytoplasm, which are similar to the eukaryotic P-bodies and stress granules, contain Ribonuclease (RNase) E and affect bacterial mRNA degradation [230]. In *Escherichia coli*, clusters of RNA polymerase (RNAP) are formed via LLPS, as cells enter the log phase in nutrient-rich media, and the transcription antitermination factor NusA forms droplets in vitro and in vivo, suggesting that this protein might nucleate formation of RNAP clusters [231]. It was also pointed out that all types of prokaryotic transcripts, such as mRNAs, rRNAs, tRNAs, and regulatory RNAs, may show distinct localizations within the cytoplasm, or the inner membrane, or the pole of rod-shaped species, with such subcellular spatiotemporal complexity and specific compartmentalization being driven by LLPS mediated by RNA-binding proteins [232].

In bacteria, FtsZ (a soluble GTPase ancestor of eukaryotic tubulin) assembles into a ring structure, termed the Z ring, precisely at the cell midpoint, where it recruits additional proteins to form the divisome, i.e., the molecular assembly that spans the membrane and that is required for cytokinesis [233]. FtsZ interacts with the nucleoid occlusion effector SlmA, a bacterial DNA-binding protein that specifically recognizes palindromic DNA sequences (referred to as SlmA-binding sequences, SBSs) and that also serves as a spatial regulator of FtsZ by antagonizing FtsZ polymerization. Interaction of FtsZ with SlmA and SBSs leads to the formation of phase-separated dynamic condensates containing FtsZ·SlmA·SBS complexes [234]. Furthermore, the dynamic distribution of bacterial division FtsZ protein is controlled by the microenvironments created by LLPS [235]. It is likely that the aforementioned examples represent the tip of the iceberg, and the future will certainly witness many more examples of bacterial MLOs and functional LLPS in bacteria.

Based on the observation that functional biomolecular condensates are present in bacteria and on the fact that (RNA) viruses replicating in the cytoplasm are capable of concentrating their replication machinery within specialized compartments, it is very likely that bacteriophages may highjack some of the bacteria-specific MLOs and biomolecular condensates and interfere with bacterial LLPS, as well. Further studies are needed to check the validity of this hypothesis. We hope that this review will foster such studies and will thereby contribute to the establishment of new concepts in the field of bacteriophage–bacteria interactions.

## 4. Implications of LLPS and MLOs for Drug Design

Based on the data herein reviewed, it is clear that viruses not only trigger LLPS mediated by their own proteins but also interfere with the biogenesis of various host MLOs, e.g., they can highjack and exploit different LLPS-based processes of the infected cells. Therefore, viral infection can be classified as a “phase separation-associated disease”. This opens some potential routes for designing novel antiviral drugs that would affect the phase-separation potential of viral proteins. Although the therapeutic means for curing phase separation-associated diseases are in general at the very early stages of their development, we agree with the envision that “the conceptual framework of LLPS provides a new paradigm for thinking about modulating protein function” and, therefore, opens new opportunities to design innovative drugs [236]. There are two major approaches that can be potentially utilized for the treatment of phase separation-associated diseases, including viral infections, which consist in targeting two different types of players engaged in biological LLPS, namely “drivers” and “controllers”. The molecules of the first type, the drivers of the separation, are specific IDPs and nucleic acids (DNA or RNA), whose interactions (protein–protein or protein-nucleic acid) are crucial for the initiation of LLPS. The molecules that act as separation controllers are often ordered proteins involved in signaling-based regulation of LLPS and/or specific enzymes controlling phase separation by catalyzing various PTMs of drivers. In fact, the known examples of PTMs capable of modulating LLPS are many, ranging from phosphorylation/dephosphorylation to acetylation/deacetylation, methylation/demethylation, and poly-SUMOylation [149,237,238,239,240,241,242,243,244]. Furthermore, aberrant PTMs can cause abnormal LLPS, associated with the pathogenesis of phase separation-associated diseases [239,245]. Furthermore, since the concentration of the drivers is the crucial factor driving LLPS, another obvious set of controllers to be targeted is that of the regulators of protein biosynthesis, degradation, and transport [236]. The involvement of such controllers in the overall protein turnover and transport makes the task of identifying the appropriate target very challenging [236]. 

On the other hand, the approach relying on targeting the drivers is also quite difficult to implement, since IDPs and IDRs are commonly considered as undruggable in the field of structure-based rational drug design. However, the accumulating evidence that, instead, disordered proteins can be efficiently targeted [246,247,248,249,250,251,252] gives hope that LLPS drivers, including viral proteins, can be targeted, as well. 

## 5. Conclusions

Data are rapidly accumulating, pointing to the ability of viruses to utilize intrinsic disorder of their own proteins to promote LLPS-driven formation of so-called “viral factories” or “viral inclusions” that are associated with viral replication and trafficking of viral components. In addition, viruses can use intrinsic disorder within either their own proteins or host proteins to hijack various cellular LLPS processes. As such, they interfere with the biogenesis of different cellular MLOs engaged in a multitude of host cell functions including those mediating the antiviral innate response. Available data so far support a scenario where viruses infecting bacterial, plant, and animal hosts are all capable of affecting LLPS and MLOs, suggesting the overall generality of this phenomenon. This virus ability represents an important but still poorly understood aspect of the host-pathogen molecular race mediated by intrinsic disorder, where new means are constantly invented by viruses to efficiently replicate inside the host cells via dysregulating, exploiting, hijacking, and invading various host molecular mechanisms. As knowledge is being gathered in this regard, we are convinced that promising and innovative antiviral strategies will emerge in a rather close future. We hope that this review will contribute to stimulating future efforts in this fascinating, emerging field.

## Abbreviations

BDV	Borna disease virus
BMC	bacterial microcompartment
DIC	differential interference contrast
EBOV	Ebola virus
EBV	Epstein–Barr virus
EPR	Electron paramagnetic resonance
ER	Endoplasmic reticulum
FRAP	Fluorescence recovery after photobleaching
FRET	Förster Resonance Energy Transfer (spectroscopy)
FUS	Fused in sarcoma protein
G3BP	Ras-GAP SH3 domain-binding protein
HeV	Hendra virus
HIV-1	Human immunodeficiency virus 1
IAV	Influenza A virus
IB	Inclusion body
IDP	Intrinsically disordered protein
IDR	Intrinsically disordered region
IFN:	Interferon
LCD	Low-complexity domain
LLPS	Liquid–liquid phase separation
MeV	Measles virus
MLO	Membrane-less organelles
MoRE	Molecular Recognition Element
MoRF	Molecular Recognition Feature
N	Nucleoprotein
NB	Negri body
NC	Nucleocapsid protein
NMR	Nuclear Magnetic resonance (spectroscopy)
PB	Processing body
PDB	Protein data bank
PLD	Prion-like domain
PreSMos	Pre-structured motif
PSE	Pre-structured element
PTM	Post-translational modification
PVA	Potato virus A
PWP	Protein waiting for a partner
RABV	Rabies virus
RNP	Ribonucleoparticle
RSV	Respiratory syncytial virus
SG	Stress granule
SFV	Semliki Forest virus
SLiM	Short linear motif
TDP-43	TAR DNA-binding protein of 43 kDa
UBP1	Oligo uridylate binding-protein 1
VSV	Vesicular stomatitis virus
ZnF	Zinc finger
